# Supplementary material for: Changing Social Mentality among University Students in the COVID-19 Pandemic: A Five-Wave Longitudinal Study in China
Source: Int J Environ Res Public Health. 2022 Mar 5;19(5):3049. doi: 10.3390/ijerph19053049 (PMC8909971; doi:10.3390/ijerph19053049)
Supplement: Supplementary file 1 [file ijerph-19-03049-s001.zip › ijerph-1596782-supplementary.pdf]

Supplementary Table S1. English version of B-DSMQ (Partly).

Guidance: Below is a list of words or phrases representing feelings, sensations, attitude, and values that people sometimes have. Please evaluate your current (or recent) life regarding psychological state from your point of view (Section 1) and assess the public's understanding of social life and social development from the perspective of the public (Section 2). Use the 6-Likert Scale below that best describes how much you have felt or experienced things this way. "1" means "very slightly or not at all", "6" means "extremely".

		**Very slightly or not at all**					**Extremely**
Section 1: Please evaluate your psychological state regarding your current life.	Composed	1	2	3	4	5	6
	Anxious and doubtful	1	2	3	4	5	6
	Unadaptable	1	2	3	4	5	6
	Meaningful	1	2	3	4	5	6
	Hopeful	1	2	3	4	5	6
	Desperate	1	2	3	4	5	6
	Optimistic	1	2	3	4	5	6
	Nervous	1	2	3	4	5	6
	Open & tolerant	1	2	3	4	5	6
	Fearful	1	2	3	4	5	6
Section 2: Please evaluate the public's understanding of social life and social development from the perspective of the public.	Rational & peaceful	1	2	3	4	5	6
	Unfair	1	2	3	4	5	6
	Disappointment	1	2	3	4	5	6
	Satisfaction	1	2	3	4	5	6
	Hostile to the rich	1	2	3	4	5	6
	Worried	1	2	3	4	5	6
	Faithful	1	2	3	4	5	6
	Gratitude	1	2	3	4	5	6
	Revenge	1	2	3	4	5	6
	Harmony	1	2	3	4	5	6

Supplementary Table S2. Post hoc analysis of differences of BSM in socio-demographic groups.

**Groups**	**Difference (95% CI)**	***p***
University		
B - A	0.76 (0.55, 0.96)	<0.001 *
C - A	0.71 (0.50, 0.91)	<0.001 *
D - A	0.94 (0.76, 1.13)	<0.001 *
E - A	0.84 (0.65, 1.03)	<0.001 *
C - B	-0.05 (-0.24, 0.14)	>0.999
D - B	0.19 (0.02, 0.36)	0.021
E - B	0.08 (-0.09, 0.25)	>0.999
D - C	0.24 (0.07, 0.41)	<0.001 *
E - C	0.13 (-0.04, 0.30)	0.308
E - D	-0.11 (-0.26, 0.04)	0.478
Major		
Art - Agriculture	-0.14 (-0.42, 0.14)	>0.999
Engineering - Agriculture	-0.23 (-0.48, 0.01)	0.085
Literature - Agriculture	-0.32 (-0.55, -0.08)	0.002 *
Medicine - Agriculture	-0.91 (-1.41, -0.41)	<0.001 *
Science - Agriculture	-0.21 (-0.45, 0.03)	0.148
Engineering - Art	-0.09 (-0.31, 0.12)	>0.999
Literature - Art	-0.18 (-0.39, 0.03)	0.179
Medicine - Art	-0.78 (-1.26, -0.29)	<0.001 *
Science - Art	-0.07 (-0.29, 0.14)	>0.999
Literature - Engineering	-0.08 (-0.24, 0.08)	>0.999

Medicine - Engineering	-0.68 (-1.15, -0.21)	<0.001 *
Science - Engineering	0.02 (-0.15, 0.19)	>0.999
Medicine - Literature	-0.60 (-1.06, -0.14)	0.002 *
Science - Literature	0.10 (-0.05, 0.26)	0.723
Science - Medicine	0.70 (0.24, 1.16)	<0.001 *
Grade		
2017 - 2016	0.10 (-0.21, 0.42)	>0.999
2018 - 2016	0.55 (0.24, 0.87)	<0.001 *
2019 - 2016	0.62 (0.31, 0.94)	<0.001 *
2018 - 2017	0.45 (0.32, 0.58)	<0.001 *
2019 - 2017	0.52 (0.39, 0.65)	<0.001 *
2019 - 2018	0.07 (-0.06, 0.20)	0.922
Parenting styles		
Neglecting - Authoritarian	-0.02 (-0.33, 0.29)	>0.999
Authoritative - Authoritarian	0.69 (0.49, 0.89)	<0.001 *
Permissive - Authoritarian	-0.27 (-0.72, 0.18)	0.694
Authoritative - Neglecting	0.71 (0.46, 0.96)	<0.001 *
Permissive - Neglecting	-0.25 (-0.72, 0.23)	>0.999
Permissive - Authoritative	-0.96 (-1.37, -0.55)	<0.001*
Harmonious Degree of Parents' Relationship		
Moderately - Not at all	0.57 (0.14, 0.99)	0.002 *
Greatly - Not at all	1.00 (0.59, 1.41)	<0.001 *
Most greatly - Not at all	1.43 (1.01, 1.85)	<0.001 *
Greatly - Moderately	0.43 (0.30, 0.57)	<0.001 *
Most greatly - Moderately	0.86 (0.71, 1.01)	<0.001 *
Most greatly - Greatly	0.43 (0.31, 0.55)	<0.001 *

*: Adjusted $P < 0.05$ after Bonferroni correction.; CI, confidence interval.; Dunn's (Bonferroni) t-test was used in post hoc analysis.

Supplementary Table S3. Standardized coefficients of independent variables on BSM in the optimal mixed model

Independent Variables	β	SE	t value	*p*
Wave	0.06	0.01	6.086	<0.001
Sex				
Male	Ref			
Female	0.159	0.056	2.84	0.005
Residence				
Others	Ref			
Shandong	0.195	0.068	2.859	0.004
Graduate				
No	Ref			
Yes	-0.519	0.178	-2.923	0.004
School				
A	Ref			
B	0.382	0.104	3.674	<0.001
C	0.493	0.1	4.917	<0.001
D	0.627	0.097	6.481	<0.001
E	0.506	0.092	5.48	<0.001
Parenting Styles				
Authoritative	Ref			
Authoritarian	-0.305	0.1	-3.034	0.002
Neglecting	-0.071	0.127	-0.557	0.578
Permissive	-0.19	0.21	-0.901	0.368

BSM indicates balanced social mentality.
